# Relationship between cardiovascular health metrics and physical performance in community-living people: Results from the Longevity check-up (Lookup) 7+ project

**DOI:** 10.1038/s41598-018-34746-4

**Published:** 2018-11-05

**Authors:** Francesco Landi, Riccardo Calvani, Anna Picca, Matteo Tosato, Emanuela D’Angelo, Anna Maria Martone, Elisabetta Serafini, Elena Ortolani, Giulia Savera, Sara Salini, Nicola Acampora, Roberto Bernabei, Emanuele Marzetti

**Affiliations:** Fondazione Policlinico Universitario “Agostino Gemelli” IRCCS, Università Cattolica del Sacro Cuore, Rome, 00168 Italy

## Abstract

Cardiovascular health metrics (CHMs) may predict disability independent of vascular events. Though, the link between CHMs and physical performance is unclear. This relationship was explored using data from the Longevity check-up (Lookup) 7+ project. Lookup 7+ is an ongoing cross-sectional survey conducted in unconventional settings across Italy. People who are at least 18-year-old and provide written informed consent are eligible. CHMs [i.e., smoking status, healthy diet, body mass index (BMI), blood pressure, blood cholesterol, and diabetes status] are assessed through closed questions and objective measurements. Physical performance is measured via the 5-repetition chair-stand test. Analyses included 7446 participants (55.5 ± 14.9 years; 56% women). Physical performance positively correlated with CHMs scores, such that participants who scored higher (6–7 points) completed the chair-stand test about 2 s faster than those scoring lower (1–2 points). In fully adjusted analysis, better physical performance was more frequently observed in younger, non-smoking, physically active men, with ideal BMI, and no diabetes. Our findings indicate a gradient of better physical function with increasing CHMs scores. Future investigations should establish the longitudinal effect of unhealthy behaviours and cardiovascular risk factors on physical performance and verify whether implementation of large-scale primordial cardiovascular prevention may positively impact physical fitness.

## Introduction

According to the Global Health and Ageing report released by the World Health Organisation (WHO), “the number of people aged 65 or older is projected to grow from an estimated 524 million in 2010 to nearly 1.5 billion in 2050, with most of the increase in developing countries”^1^. This demographic transition poses tremendous challenges for managing the care of older individuals, such that the maintenance of independence in advanced age is considered a top public health priority^2^.

Despite the prevention strategies adopted in the last decades and the development of more effective therapeutic options, cardiovascular disease (CVD) remains the leading cause of morbidity, disability, and mortality, both in Europe and in the United States^3^. A step forward for reducing the burden of CVD at the population level entails careful anticipation of risk factors through primordial prevention^4^. To this aim, the American Heart Association (AHA) “Strategic Impact Goal Through 2020 and Beyond” defined the metrics that may be used to describe cardiovascular health in the population^5^. The cardiovascular health metrics (CHMs) proposed by the AHA consist in the combination of four health behaviours [smoking, diet, physical activity, and body mass index (BMI)] and three health factors (blood pressure, total blood cholesterol, and blood glucose)^5^. The promotion of cardiovascular health through these metrics is the cornerstone of the “Life’s Simple 7” programme (i.e., manage blood pressure, control cholesterol, reduce blood sugar, get active, eat better, lose weight, stop smoking)^6^.

People who meet a greater number of ideal CHMs show lower prevalence and incidence of both CVD and non-cardiovascular conditions, such as cancer, depression, and cognitive impairment^7^. Notably, CHMs have been shown to predict physical disability and declining physical performance independent of vascular events in middle-aged persons and older adults^8–11^. However, the relationship between CHMs and physical function across ages is still unclear.

The present study was, therefore, undertaken to explore the relation between CHMs and physical performance in a large and unselected sample of community-living people across a wide age spectrum enrolled in the Longevity check-up (Lookup) 7+ project. Physical performance was elected as the main exposure of interest given the ability of physical function-related measures to predict relevant health outcomes, including disability and mortality, in young and old adults^12–14^.

## Results

For the present study, 7446 participants were considered, after excluding 351 people for missing blood cholesterol and/or glucose values and 243 for missing 5-repetition chair-stand test values. Age and gender distribution of participants not included in the analyses were comparable to those of the remaining enrolees. The main characteristics of the study population according to gender are summarised in Table 1. The mean age of participants was 55.5 years [standard deviation (SD), 14.9 years; range: 18–98 years], with 4199 (56%) women. As compared with men, women showed higher CHMs summary scores (4.4 ± 1.3 vs. 4.0 ± 1.3; p < 0.001). In particular, women had higher prevalence of non smoking (p < 0.005), healthy diet (p < 0.001), ideal BMI (p < 0.001) and blood pressure (p < 0.001), and absence of diabetes (p < 0.001). Conversely, men were more frequently physically active (p < 0.001) and had a higher prevalence of ideal cholesterol levels than women (p = 0.001). Overall, performance on the chair-stand test was better among men (7.7 ± 2.1 s vs. 8.0 ± 2.4 s; p < 0.001).

**Table 1:** Characteristics of the study population according to gender*.

Characteristics	Total sample (n = 7446)	Men (n = 3247)	Women (n = 4199)	p value
Age (years)	55.5 ± 14.9	55.5 ± 15.2	55.4 ± 14.7	0.71
Non-smoking	6191 (83)	2658 (82)	3533 (84)	0.005
Healthy diet ^§^	5284 (71)	2162 (66)	3122 (74)	<0.001
Physically active ^§^	4138 (55)	1926 (59)	2212 (53)	<0.001
Ideal body mass index ^§^	3605 (48)	1189 (37)	2416 (57)	<0.001
Ideal blood pressure ^§^	3114 (42)	1051 (32)	2063 (66)	<0.001
Ideal total blood cholesterol ^§^	2412 (32)	1113 (34)	1299 (31)	0.001
No diabetes	6869 (92)	2948 (91)	3921 (93)	<0.001
Cardiovascular health metrics score	4.2 ± 1.3	4.0 ± 1.3	4.4 ± 1.3	<0.001
5-repetition chair-stand test (s)	7.9 ± 2.3	7.7 ± 2.1	8.0 ± 2.4	<0.001

^*^Data are given as number (percent) for all variables, except for age, cardiovascular health metrics score, and 5-repetition chair-stand test that are shown as mean ± standard deviation.

^§^Healthy diet: consumption of at least three portions of fruit and/or vegetables per day; physically active: physical activity at least twice weekly during the past year; ideal body mass index: 18.5–24.9 kg/m^2^; ideal total blood cholesterol: < 5.18 mmol/L (200 mg/dL), untreated; ideal blood pressure: < 120/80 mmHg, untreated.

Predictive factors associated with better performance on the chair-stand test (<7.75 and <7.43 s for women and men, respectively) are shown in Table 2. In the adjusted model, older age was negatively associated with physical performance, indicating that older people were more likely to take longer to complete the test. Remarkably, non smoking [odds ratio (OR) 1.29, 95% confidence interval (CI) 1.12–1.48], being physically active (OR 1.37, 95% CI 1.23–1.52), having ideal BMI (OR 1.46, 95% CI 1.31–1.62), and being free of diabetes (OR 1.22, 95% CI 1.01–1.48) were significantly associated with better performance.

**Table 2:** Unadjusted and adjusted association [odds ratio (OR) and 95% confidence intervals (CIs)] between cardiovascular health metrics and performance on the 5-repetition chair-stand test better than gender-specific median values (i.e., <7.75 and <7.43 s for women and men, respectively).

	Unadjusted Odds Ratio (95% CI)	Adjusted Odds Ratio* (95% CI)
Age	0.94 (0.93–0.94)	0.94 (0.93–0.95)
Gender (male)	1.26 (1.15–1.38)	1.46 (1.31–1.62)
Non smoking	1.15 (1.00–1.32)	1.29 (1.12–1.48)
Healthy diet	1.28 (1.15–1.41)	1.01 (0.90–1.14)
Physical activity	1.40 (1.27–1.53)	1.37 (1.23–1.52)
Ideal body mass index	1.88 (1.72–2.06)	1.46 (1.31–1.63)
Ideal blood pressure	2.00 (1.87–2.19)	1.07 (0.96–1.20)
Ideal total blood cholesterol	1.21 (1.10–1.34)	0.83 (0.74–1.01)
No diabetes	1.99 (1.67–2.37)	1.22 (1.01–1.48)

^*^Simultaneously adjusted for age, gender, and the seven cardiovascular health metrics.

**Figure 1:**
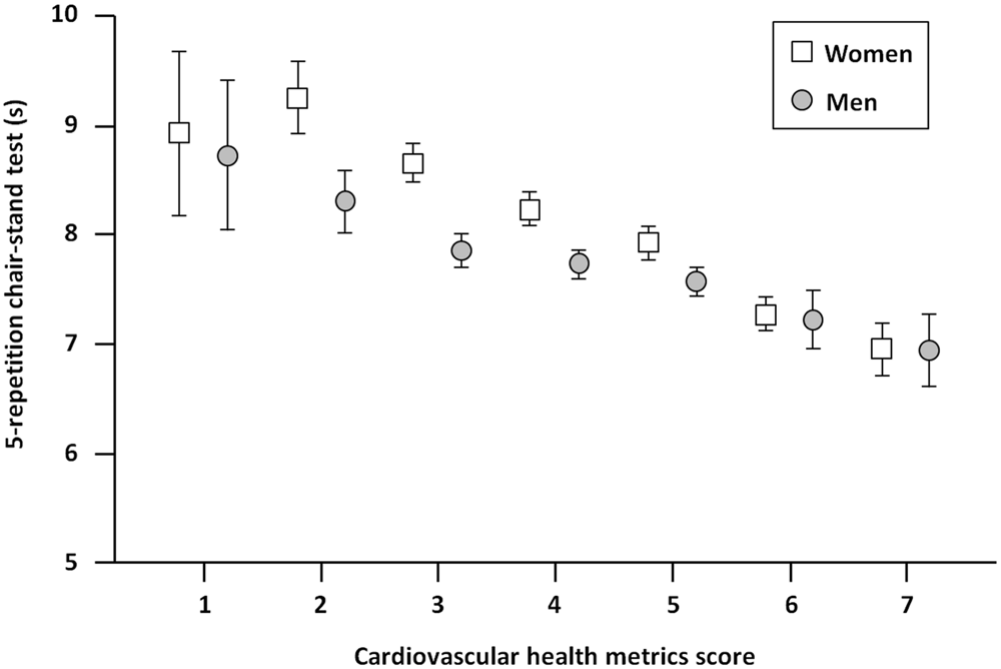
Time to complete the 5-repetition chair-stand test according to cardiovascular health metrics score and gender. In both genders, the time needed to complete the chair-stand test is inversely related to the cardiovascular health metrics score, with the worst and best performance observed in participants with scores of 1–2 and 6–7, respectively (p for trend < 0.001).

Results of age-adjusted analysis of covariance (ANCOVA) of chair-stand performance across CHMs scores are depicted in Fig. 1. A significant different distribution of performance across CHMs scores was observed (p for trend <0.001). The time to complete the test was similar for CHMs score of 1 and 2 points, and declined linearly across better scores. Specifically, comparing worse (1–2 points) versus better scores (6–7 points), the time to complete the test was 2.0 s higher in men (8.9 s vs. 6.9 s; p < 0.001) and 1.7 s higher in women (8.7 s vs. 7.0 s; p < 0.001).

**Figure 2:**
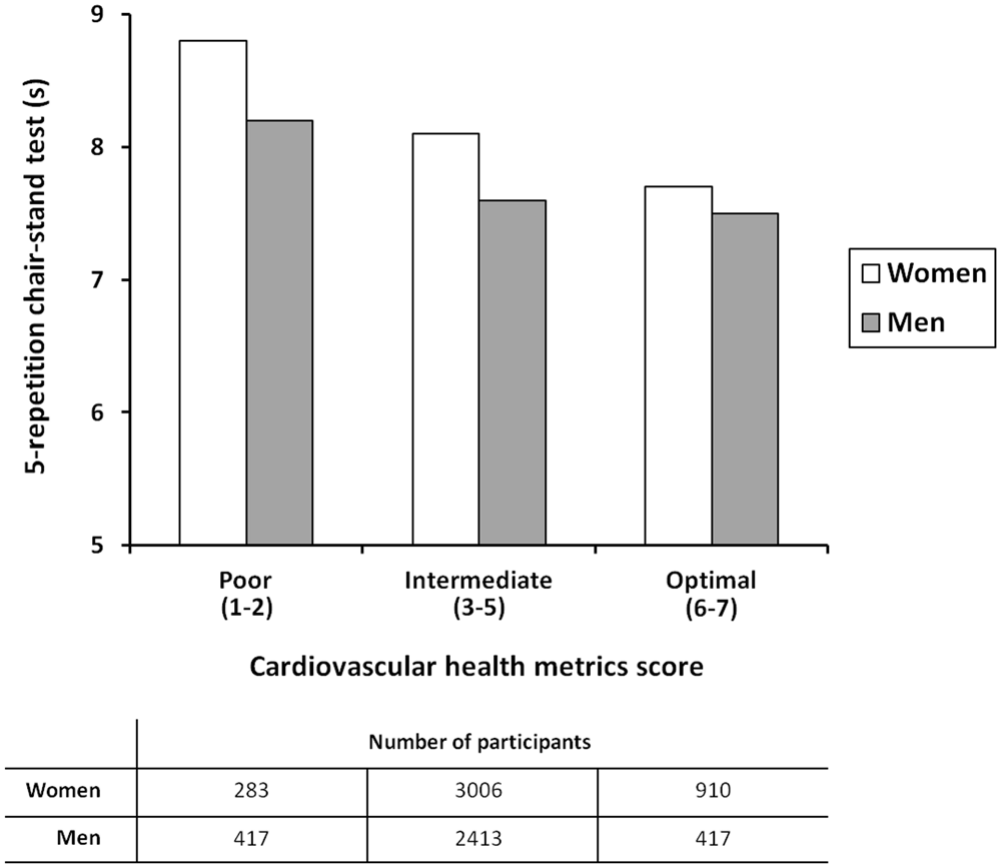
Age-adjusted performance on the 5-repetition chair-stand test across categories of cardiovascular health metrics scores. In both genders, the time needed to complete the chair-stand test is lower with better categories of cardiovascular health metrics scores (p for trend <0.001).

Figure 2 shows the age-adjusted performance on the chair-stand test across categories of CHMs scores. In both genders, the time taken to complete the test followed a downward gradient across poor, intermediate and optimal CHMs scores (p for trend <0.001).

## Discussion

In the present study, established CHMs were assessed in a large and relatively unselected cohort of Italian community-dwelling men and women ranging in age between 18 and 98 years. Physical performance was quantified to obtain a more comprehensive characterisation of participant health status. It should be noted that the Lookup 7+ project offered the unique opportunity of assessing these domains in people of both genders, across a wide age range, outside of conventional healthcare or research settings. Using this innovative database, the present study shows that, independent of age and gender, better physical performance is more frequently observed among non-smoking, physically active participants, with ideal BMI, and free of diabetes. Furthermore, a linear correlation was determined between CHMs scores and the time taken to complete the chair-stand test. Specifically, a gradient of better physical performance with increasing CHMs scores was found.

Few studies explored the association between CHMs and physical function. In the Northern Manhattan Study, higher CHMs scores were associated with better functional status, as assessed by the Barthel index^15^, among 3219 community-living adults aged 40 + over 10 years of follow-up^8^. The association remained significant after accounting for incident cardiovascular events or when mobility and non-mobility domains of the Barthel index were analysed separately^8^. A cardiovascular health index based on the CHMs proposed by the AHA has shown to predict the risk of declining physical function, as assessed through the short physical performance battery (SPPB)^12^, in older men and women enrolled in the inCHIANTI study^11^. Of the three SPPB subtasks (i.e., balance, gait speed, and chair-stand test), the cardiovascular health index was more strongly related to lower extremity muscle power and walking speed^11^. A similar index has been associated with handgrip strength in a subsample of more than 25,000 late middle-aged men and women of the UK Biobank study after adjustment for several potential confounders^16^. Notably, CHMs assessed in mid-life predicted SPPB scores 25 years later in 6144 participants of the Atherosclerosis Risk in Communities (ARIC) Study^17^. Independent associations between CHMs and measures of physical fitness have also been determined in healthy children and adolescents^18,19^ as well as in specific patient populations^20^. The relevance of cardiovascular health to physical function and overall health is further substantiated by the recent association determined between CHMs and the frailty status^16,21^. Indeed, Graciani and colleagues showed that meeting a higher number of ideal CHMs conferred reduced risk of developing frailty among 1745 people aged 60 years or older over a 3.5-year follow-up^21^. The association remained significant after accounting for incident CVD. Similarly, among 421,000 community-dwellers aged 60 to 69 years, those with near-ideal cardiovascular risk factor profile showed lower risk of becoming frail during up to 10 years of follow-up^16^. It is noteworthy that, within the same time-frame, participants with better cardiovascular risk profile had lower incidence of incontinence, falls, fragility fractures, and dementia^16^.

Numerous mechanisms may be invoked to explain the relationship between cardiovascular risk factors and physical performance. First, participants with lower CHMs scores might have experienced previous cardiovascular events that impacted their fitness. However, in the NHANES study, CHMs were not predictive of mobility disability among participants with previous stroke and/or myocardial infarction^9^. This may suggest that, once an individual develops clinically manifest CVD, risk factors captured by CHMs might be unable to predict additional functional impairment. On the other hand, CHMs components may be associated with accrual of subclinical vascular disorders that impact physical performance before clinically evident CVD ensues^9^. For instance, neuroimaging studies showed that subclinical white matter lesions and small-size brain infarcts were associated with reduced performance on the chair-stand test in community-dwelling older adults^22^. Furthermore, endothelial dysfunction was associated with poorer muscle power of the lower extremities in a sample of community-living elderly^23^.

Taken together, the results of the present study along with previous findings on the subject suggest the possibility of promoting physical fitness through primordial cardiovascular prevention. As forecasted by the AHA, the implementation of nation-wide primordial cardiovascular prevention would result in 20% reduction of CVD burden by the year 2020^5^. Since CHMs predict functional impairment independent of vascular events, it is plausible that the preservation of functional status through primordial prevention strategies might reduce the burden associated with poor physical function well beyond 20%^9^.

Albeit dealing with a highly relevant issue, our study presents several limitations that need to be discussed. First, results were obtained from a cross-sectional survey; hence, cause-effect relationships between CHMs and physical performance may not be inferred. Random cholesterol and glucose determinations could lead to overestimating both parameters. Conventionally, blood samples for lipid analysis are drawn in the fasting state. However, fasting and non-fasting sampling gives similar results for total cholesterol, low-density lipoprotein-cholesterol and high-density lipoprotein-cholesterol^24^. Cholesterol and glucose were measured in capillary blood samples. Although the procedure was previously validated^25^, the error of portable devices is higher than with standard equipment. The type of evaluation and its setting could have influenced the assessment of some health metrics and physical performance. Indeed, people who decided to participate were involved – before being assessed – in usual exhibition and/or shopping centre activities, such as walking, carrying bags, and eating, which could have influenced the assessment. In particular, the chair-stand test in older individuals might have been more affected by tiring activities than in younger participants. Furthermore, alcohol and coffee drinking, which may affect blood pressure and blood lipids levels, was not recorded. In addition, no information was collected about pre-existing cardiovascular or non-cardiovascular conditions, which prevented us from adjusting the analyses for these potential confounders. The questionnaire used for estimating physical activity, although developed based on information collected during similar surveys^26–32^, was not formally validated. As illustrated in the methods section, the definition of ideal categories adopted for some CHMs differs from that developed by Lloyd-Jones *et al*.^5^ This may limit comparison with studies that have used the original definition. Finally, the Lookup 7+ population included only Caucasian persons; therefore, our results may not be applicable to other ethnic groups.

Despite these intrinsic limitations, the Lookup 7+ project offered the unique opportunity to investigate the relationship between CHMs and physical performance in a large sample of “real-world” people across a wide age range. The association between optimal cardiovascular health and better physical performance suggests the existence of shared mechanisms linking CVD and functional status. Further studies are needed to establish the longitudinal impact of unhealthy behaviours and cardiovascular risk factors on physical performance and to verify whether the implementation of large-scale primordial cardiovascular prevention is effective at improving physical fitness.

## Methods

### Study population

Data used for the present study were collected as part of the Lookup 7+ project, an ongoing initiative developed by the Department of Geriatrics of the Università Cattolica del Sacro Cuore (Rome, Italy). As previously described^30^, the project started on June 1^st^, 2015 and was designed to promote the adoption of healthier lifestyles by raising awareness in the general population on major lifestyle behaviours and risk factors for chronic diseases. A team of medical doctors, researchers, and nutritionists assessed people visiting public places (e.g., malls, exhibition centres) and those adhering to prevention campaigns launched by our department. This approach was chosen because allowing enrolment of relatively unselected participants, outside of conventional healthcare or research settings.

The assessment protocol has been described in detail elsewhere^26^. Candidate participants are considered to be eligible for enrolment if they are at least 18 years of age and provide written informed consent. Unwillingness to give written informed consent, pregnancy, inability to perform functional tests, and refusal of blood capillary check are considered exclusionary. The study was conducted according to the principles expressed in the Declaration of Helsinki and the protocol was approved by the Università Cattolica del Sacro Cuore Ethics Committee (protocol #: A.1220/CE/2011).

Analyses were conducted in participants enrolled between June 1^st^, 2015 and December 31^st^, 2017, in the following settings: *Milan EXPO 2015* (Milan, June-October 2015), *Mese del Cuore* (Rome, September-October 2016 and September-October 2017; Milan, March-April 2017), *Ministry of Health – Women’s Day* (Rome, April 2017), *CamBio Vita* (Catania, May 2017), *COOP shopping centres* (Bologna, Modena, Genoa, Rimini, and Grosseto, May-June 2017), *Tennis & Friends* (Rome, October 2017), *CONAD shopping centres* (Terni, Perugia, Viterbo, Anzio, Caserta, November 2017), and *La Romanina – Check your Longevity* (Rome, December 2017).

Depending on the setting, the initiative was advertised in newspapers, magazines and TV broadcasting. Visitors were also invited to participate by direct contact. All participants who accepted to participate in the Lookup 7+ initiative received a dedicated assessment that was conducted in a setting ensuring the utmost respect for privacy. The evaluation comprised a lifestyle interview (smoking and eating habits, habitual physical activity), blood pressure measurement, weight and height assessment, glucose and total blood cholesterol measurements, and the chair-stand test for the assessment of physical performance^26,31^. As previously reported, each Lookup 7+ assessment required 13.2 ± 1.7 min to be completed (range: 9.1–17.8 min)^30^. Although study participants or public were not formally involved in the design of the study, the questionnaire used for data collection and the specific assessments conducted were developed based on the information collected during similar surveys^26–31,33^. It is noteworthy that, as reported elsewhere^30^, over 95% of Lookup 7 + participants declared being either satisfied or very satisfied with the evaluation.

### Assessment of cardiovascular health metrics

The definition of ideal CHMs as originally proposed by Lloyd-Jones *et al*.^5^ and the corresponding definitions adopted in the Lookup 7+ study are presented in Table 3. Smoking status was defined as follows: current smoker (has smoked 100+ cigarettes in lifetime and currently smokes cigarettes), never smoked (has never smoked or has smoked <100 cigarettes in lifetime), and former smoker (has smoked at least 100 cigarettes in lifetime but had quit at least 28 days before the interview)^30^. For the purpose of the analyses, smoking status was categorised as current or never/former smoker. Healthy diet was considered as the consumption of at least three portions of fruit and/or vegetables per day^28^. For the calculation of daily intake of fruit and vegetables, reference tables for the Italian population released by the Italian Society of Nutrition (SINU) were used. Accordingly, three or more portions of fruit and/or vegetables correspond to more than 400 g, which is the minimum amount recommended by the WHO^34^. The use of three or more portions to identify a healthy diet is in line with Italian dietary habits for fruit and vegetables which are typically eaten during the main meals rather than as snacks. Reference amounts are available at http://www.sinu.it/html/cnt/larn.asp. Regular participation in physical activity was considered as involvement in leisure-time physical activity at least twice a week during the past year^31^. Accordingly, participants were considered either physically inactive or active. To be assigned to the latter group the following activities were considered: brisk walking for at least 30 min per session or cycling, swimming, running, and resistance training for at least 20 min per session^27,31^. Body weight was measured through an analogue medical scale, while a standard stadiometer was used to measure height. BMI was subsequently calculated as weight (kg) divided by the square of height (m). Blood pressure was measured with a clinically validated Omron M6 electronic sphygmomanometer (Omron, Kyoto, Japan), according to recommendations from international guidelines^35^. Total cholesterol was measured from capillary blood samples using disposable electrode strips based on a reflectometric system with a portable device (MultiCare-In, Biomedical Systems International Srl, Florence, Italy)^25^. The same device was employed to measure random blood glucose using disposable reagent strips based on an amperometric system^25^. Those who declared being diabetic and, according to international guidelines^36^, those who presented with a random blood glucose level ≥11.1 mmol/L (200 mg/dL) were considered to be diabetic.

**Table 3:** Definition of ideal cardiovascular health metrics in the Lookup 7+ study and the American Heart Association “Strategic Impact Goal Through 2020 and Beyond”^5^.

Cardiovascular health metrics	Definition of ideal metrics	
	Lookup 7+	American Heart Association^*^
Smoking status	Never smoked or quit ≥28 days before the interview	Never smoked or quit >12 months before the interview
Healthy diet	≥ 3 portions of fruit and/or vegetables per day	4–5 components of the American Heart Association healthy diet
Physical activity	Involvement in physical activity at least twice a week during the past year^§^	≥150 min/week of moderate intensity activity or ≥75 min/week of vigorous intensity activity or their combination
Body mass index	18.5–24.9 kg/m^2^	<25 kg/m^2^
Blood pressure	<120/80 mmHg	<120/80 mmHg
Total blood cholesterol	<200 mg/dL	<200 mg/dL
Blood glucose	Absence of diabetes	Fasting plasma glucose <100 mg/dL

^*^Definitions for adults >20 years of age.

^§^Brisk walking for at least 30 min per session or cycling, swimming, running, and resistance training for at least 20 min per session.

The following findings were considered as ideal CHMs: never/former smoker, regular engagement in physical activity, healthy diet, BMI 18.5–24.9, untreated blood pressure <120/80 mmHg, untreated total blood cholesterol <5.18 mmol/L (200 mg/dL), and absence of diabetes. The latter criterion was chosen in place of the standard definition of fasting blood glucose <5.6 mmol/L (100 mg/dL) because glycaemia was determined in random capillary blood samples. This approach has proven to be suitable for the screening of non-hospitalised populations^37^. One point was assigned to each ideal metric, while a score of 0 was attributed to non-ideal categories. The CHMs score was finally calculated as the sum of individual items (range 0–7)^5^.

### Assessment of physical performance

Physical performance was evaluated via the 5-repetition chair-stand test, one of the subtasks of the SPPB^12^. Previous studies have documented that the chair-stand test is highly reliable [intraclass correlation coefficients (ICCs) 0.76–0.99 for test-retest reliability; ICCs 0.97–1.00 for inter-rater reliability] in adult and older persons^38,39^. In advanced age, poor performance on this test is associated with mobility difficulties and is correlated with incident disability^12,40,41^. The chair-stand test also provides information on physical fitness and muscle strength across a wide age range^42^. Furthermore, timed chair-stand tests may be used as a quick measurement of functional capacity in healthy young people^43^. The test is especially suitable for the settings in which the Lookup 7+ project takes place because it is easily and quickly administered and only requires a standard armless chair and a stopwatch.

During the Lookup 7+ assessment, participants were thoroughly instructed on the requirement of the test by staff members who had been previously trained and certified at the Department of Geriatrics of the Università Cattolica del Sacro Cuore. Following a single-repetition demonstration by the assessor, the participant was invited to stand up once from the chair with his/her arms folded across the chest. After the movement was performed correctly, the participant was asked to stand up from the chair five times in a row as quickly as possible. A standard armless chair, 43–47 cm in height was used. The chair’s back was placed against a wall to ensure safety and stability. The time taken to complete the task was measured using a handheld stopwatch, with longer time reflecting poorer performance. For the purpose of analysis, participants were classified in two groups according to gender-specific chair-stand test median values (7.75 and 7.43 s for women and men, respectively).

### Statistical analyses

Continuous variables are expressed as mean ± SD, whereas categorical variables are given as absolute numbers and percentages. Descriptive statistics were used to assess demographics and main clinical characteristics of the study population according to gender. Differences in proportions and means of covariates between genders were assessed using the Fisher’s exact test and the t-test, respectively.

Logistic regression analysis was used to explore the association between CHMs and the performance on the chair-stand test. To identify factors independently associated with better performance (higher than gender-specific median values), we first estimated the crude prevalence rate ratio at a 95% CI, and then controlled for age and gender. A logistic regression analysis was computed including simultaneously the seven CHMs. ANCOVA – adjusted for age – was used to examine the effect of CHMs summary score on the chair-stand test. CHMs scores were also categorised as poor (1–2), intermediate (3–5), and optimal (6–7). Age-adjusted ANCOVA was used to determine the relationship between CHMs score categories and the results of the chair-stand test. All analyses were computed using the SPSS software (version 18.0, SPSS Inc., Chicago, IL).

## Data Availability

Data analysed in the current study are available from the corresponding author on reasonable request.
